# Comparison of clinicopathological characteristics of lymph node positive and lymph node negative breast cancer

**DOI:** 10.12669/pjms.324.10324

**Published:** 2016

**Authors:** Naila Irum Hadi, Qamar Jamal

**Affiliations:** 1 Dr. Naila Irum Hadi, MPhil, PhD Fellow. Department of Pathology, Ziauddin University, Karachi, Pakistan; 2 Dr. Qamar Jamal, MPhil, PhD. Department of Pathology, Ziauddin University, Karachi, Pakistan

**Keywords:** Breast cancer, Clinicopathological characteristics, Histological grade, Nodal metastasis, TNM stage, Tumor size

## Abstract

**Objective:**

To record various clinicopathological characteristics of breast cancer (BC) in our population and to find an association between these characteristics and axillary nodal metastasis.

**Methods:**

This cross-sectional study included 150 BC patients from two tertiary care centers in Karachi from 15^th^ February, 2013 to 31^st^ March, 2015. Frequencies, percentages, and odds ratio were estimated to find out an association between various clinicopathological characteristics and lymph node status using SPSS version 20.

**Results:**

Approximately 75.4% patients had axillary lymph node metastasis (‘1-3’ LN = 34.4% and ‘>3’ LN = 44%). Menopausal status (p <0.013), tumor grades (‘II’ p <0.03; ‘III’ p <0.01), and stages (‘III’ p <0.002; ‘IV’ p <0.0001), tumor sizes (‘T2’ p <0.014; ‘T3’ p <0.002), perineural invasion (PNI) (p <0.007), lymphovascular invasion (LVI) (p <0.0001), and skin and nipple invasion (p <0.024) were significant predictors for ‘>3’ LN metastasis. Association of these variables with ‘1-3’ LN involvement was insignificant.

**Conclusion:**

Clinical spectrum of BC remains unchanged in 2016 with most of the patients presenting with high-grade, late-stage advanced disease. Moreover, clinicopathological variables, especially primary tumor size, tumor stage and lymphovascular invasion were significant predictors of >3 lymph node metastasis with high accuracy.

## INTRODUCTION

Pakistan has the highest incidence of breast cancer (BC) among Asian women.^1^ Mean age for BC at the time of diagnosis falls in the 4^th^ & 5^th^ decade and the affected women mostly presented at an advanced stage with loco regional spread.^1^-^3^ More than 95% of malignant tumors of breast are adenocarcinomas with infiltrating ductal carcinoma being the most common type (40% - 80% of all invasive cancers).^1^,^2^

Axillary lymph node (LN) involvement in BC has diagnostic, prognostic as well as therapeutic significance.^4^ In 1978, the Union for International Cancer Control (UICC) and American Joint Committee on Cancer (AJCC) developed TNM (“Tumor, node, metastasis”) staging system, which is commonly used for BC. It has been periodically updated with the seventh edition completed in 2010.^5^ The ‘N’ in TNM staging (AJCC) denotes axillary lymph node involvement, and groups BC patients into three ‘N’ stages: N1 (1 to 3 LNs positive), N2 (4 to 9 LNs positive), N3 (more than 9 LNs positive). Isolated tumor cells or minimal tumor involvement is denoted as N0.^5^

Tumor size correlates with number of lymph nodes involved; the larger the tumor size, worst is the prognosis. Moreover in node-negative BC, tumor size becomes the most significant prognostic factor for decisions regarding adjuvant therapy.^6^ Prognostic significance for histologic grading as well as lymphovascular invasion (LVI) is limited to node-negative breast cancers, with borderline tumor sizes.^7^

The presence of ER, PR, or HER2/neu in BC predicts the response of tumor to anti-estrogen (tamoxifen) or Herceptin (trastuzumab) therapy. Gaopande et al. (2015)^8^ found triple-negative BC (TNBC) in younger women, with higher grade, larger size, and lymphovascular invasion. He et al. (2015)^9^ divided invasive intraductal BC into four different subtypes on the basis of ER, PR and HER2 expression and studied the association between different BC subtypes and axillary lymph node involvement.

These histopathologic features have significant prognostic information for breast cancer^3^,^8^,^10^ and have been described as predictors for axillary lymph node metastasis.^9^,^11^ However limited data^9^,^12^,^13^ is available for Asian countries including Pakistan, so significance of these variables needs to be assessed in our population.

Hence the purpose of the present study was first to evaluate the clinicopathological features of breast cancer and then to compare these in lymph node positive and negative BC patients from two tertiary care centers in Karachi, Pakistan.

## METHODS

A total of 150 BC patients were included in this prospective study using the non-probability sampling technique. Only patients with invasive breast cancer with modified mastectomies and axillary lymph node resection from Ziauddin University Hospital and Jinnah Postgraduate Medical Center, Karachi were included. The study period was from February 2013 to March 2015 and it was approved by Ethics Review Committee of Ziauddin University, Karachi. Informed written consent was obtained from all the patients and confidentiality of their personal details was maintained.

Gross examination was done according to standard College of American Pathologists (CAP) protocol with special emphasis on tumor size, skin, nipple and lymph node involvement and confirmed later with histopathological examination. Histological grading (grade I to III) and staging (stage I to IV) were done according to Modified Scarff-Bloom-Richardson (SBR) grading system and TNM staging respectively. Estrogen receptor (ER), progesterone receptor (PR) and human epidermal growth factor receptor (HER2/neu) status were determined by immunohistochemistry. In this study, HER2/neu score of 3+ and 2+ was recorded as positive and 1+ as negative.

Information regarding lymph node involvement included total number of lymph nodes recovered, number of lymph nodes showing metastatic deposits and extracapsular involvement. BC cases were stratified by tumor size (T1, <2 cm; T2, 2-5 cm; T3, >5 cm) 3,13 and nodal status (‘0’ LN; ‘1-3’ LN; ‘>3’ LN).^3^

Data was analyzed in Statistical Package for Social Sciences (SPSS, version 20, IBM). Frequencies and percentages were computed and an association between nodal status and clinicopathological variables was seen by Chi-square and Fisher’s exact probability tests. Two-tailed p value less than 0.05 was considered statistically significant. Odds ratio (OR) with corresponding 95% confidence interval (CI) predicting nodal metastasis were also estimated.

## RESULTS

Tumor characteristics and histopathological features of BC are shown in Table-I. Overall 150 lymph node positive and negative BC patients were included with mean age of 46.7 years ± 11.68 (95% CI 44.8 – 48.6; age range 22 – 76 years). More than half of the patients (66.9%) were younger than 50 years at presentation (mean age 40.2 ±years ±6.82; 95% CI 38.9 – 41.5; range 22 – 50 years). Most of the tumors were invasive ductal carcinoma (IDC) (140; 93.3%) with only 7 cases (4.7%) of invasive lobular carcinoma (ILC), two cases (1.3%) of metaplastic breast cancer (MBC) and one case (0.67%) of mucinous carcinoma.

**Table-I:** Patient and tumor characteristics by Lymph node involvement in Breast Cancer.

Characteristics	Total Patients n (%)	Lymph Node Involvement n (%)			P value

	150	‘0’ LN (37)	’1-3’ LN (47)	’>3’ LN (66)	
***Menopausal status***					
Premenopausal	72 (48)	13 (18.1)	18 (25)	41 (56.9)	
Postmenopausal	78 (52)	24 (30.8)	29 (37.2)	25 (32.1)	0.009
***Histological Grade***					
Grade I	12 (8)	4 (33.3)	4 (33.3)	4 (33.3)	
Grade II	71 (47.3)	20 (28.2)	22 (31)	29 (40.8)	0.66
Grade III	67 (44.7)	13 (19.4)	21 (31.3)	33 (49.3)	
***Tumor Stage***					
Stage I	6 (4)	5 (83.3)	1 (16.7)	0 (00)	
Stage II	40 (26.7)	19 (47.5)	20 (50)	1 (2.5)	0.0001
Stage III	85 (56.7)	11 (12.9)	20 (23.5)	54 (63.5)	
Stage IV	19 (12.7)	2 (10.5)	2 (10.5)	11 (57.9)	
***Tumor Size***				
< 2 cm	18 (12)	10 (55.6)	3 (16.7)	5 (27.8)	
2-5 cm	79 (52.7)	19 (24.1)	32 (40.5)	28 (35.4)	0.001
> 5 cm	53 (35.3)	8 (15.1)	12 (22.6)	33 (62.3)	
***Distant Metastasis***					
Absent	126 (84)	36 (27.5)	40 (30.5)	50 (38.2)	0.253
Present	19 (13.1)	1 (5.3)	7 (36.8)	11 (57.9)	
***Lymphovascular Invasion***					
Absent	97 (64.7)	31 (32)	35 (36.1)	31 (32)	0.0001
Present	53 (35.3)	6 (11.3)	12 (22.6)	35 (66)	
***Perineural Invasion***					
Absent	137 (91.3)	37 (27)	45 (32.8)	55 (40.1)	0.007
Present	13 (8.7)	0 (00)	2 (15.4)	11 (84.6)	
***Skin & Nipple Invasion***					
Absent	92 (61.3)	28 (30.4)	29 (31.5)	35 (38)	0.055
Present	37 (24.7)	4 (10.8)	13 (35.1)	20 (54.1)	
***Hormone Receptors & HER2***					
ERPR+/HER2-	54 (36)	13 (24.1)	20 (37)	21 (38.9)	0.245
ERPR+/HER2+	25 (16.7)	5 (20)	5 (20)	15 (60)	0.327
ERPR-/HER2+	25 (16.7)	4 (16)	9 (36)	12 (48)	0.66
ERPR-/HER2-	30 (20)	7 (23.3)	7 (23.3)	16 (53.3)	0.612

LN, lymph node; Vs, versus; n, number of patients; OR, odds ratio; CI, confidence interval; ER, estrogen receptor; PR, progesterone receptor; HER2, human epidermal growth factor receptor 2.

There were 37 (24.7%) patients with no LN involvement, 47 (31.4%) with 1-3 LNs and 66 (44%) with >3 LNs involved by tumor. Extracapsular extension was noted in 80 (53.3%) cases. The minimum number of affected lymph nodes recovered per case was one and maximum number was 45 (95% CI 44.3 – 45.7).

According to histological grading and TNM staging, most of the cases were high grade (II & III = 92%) and late stage tumors (II & III = 83.4%) (Table-I). Tumor size ranged from 0.2 cm to 19.5 cm (mean 4.94 ± 3.4; 95% CI 4.4 – 5.5). Univariate analysis revealed that menopausal status (p=0.009), tumor stage (p=0.0001), tumor size (p=0.001), LVI (p=0.0001) and PNI (p=0.007) were significantly associated with nodal status (Table-I). Multivariate analysis was done to compare the different clinicopathological characteristics with lymph node involvement (‘0’ LN Vs ‘1-3’ LN & ‘0’ LN Vs ‘>3’ LN). Menopausal status, tumor grade and tumor stage, tumor size, LVI, PNI and skin and nipple invasion were significant predictors for ‘>3’ LN involvement with highest odds ratio reported for tumor stage, tumor size and LVI respectively (Table-II).

**Table-II:** Odds Ratio (OR) for Clinicopathological Predictors of Nodal Status in BC.

		‘1-3’ LN			‘> 3’ LN	

Characteristics	OR	95% CI	P Value	OR	95% CI	P Value
***Menopausal Status***						
Premenopausal Vs Postmenopausal	0.873	0.36-2.14	0.822	0.33	0.14-0.76	0.013
***Tumor Size (cm)***						
2-5 Vs <2	2.95	0.87-9.99	0.136	5.614	1.37-22.98	0.014
>5 Vs <2	5	1.04-24.03	0.072	8.25	2.19-30.96	0.002
>5 Vs <5	1.243	0.45-3.45	0.798	3.625	1.45-9.09	0.006
***Tumor Grade***						
II Vs I	1.467	0.29-7.37	0.702	0.833	0.69-0.99	0.036
III Vs I	2.154	0.41-11.2	0.421	0.765	0.58-0.99	0.01
***Tumor Stage***						
II Vs I	5.263	0.56-49.29	0.193	0.792	0.65-0.97	1
III Vs I	9.091	0.94-87.96	0.066	24.09	2.56-226.9	0.002
IV Vs I	36	1.8-718.7	0.029	0.143	0.02-0.88	0.0001
***Lymphovascular Invasion***						
Present Vs Absent	1.771	0.59-5.28	0.423	5.833	2.15-15.84	0.0001
***Perineural Invasion***						
Present Vs Absent	1.044	0.98-1.11	0.501	1.2	1.17-1.34	0.007
***Skin & Nipple Invasion***						
Present Vs Absent	3.138	0.91-10.79	0.094	4	1.23-13.06	0.024
***Distant Metastasis***						
Present Vs Absent	3.063	0.59-15.72	0.287	3.5	0.73-16.74	0.128
***Hormone Receptors & HER2***						
ERPR+/HER2- Vs ERPR-/HER2-	0.733	0.19-2.89	0.734	1.375	0.42-4.56	0.765
ERPR+/HER2+ Vs ERPR-/HER2-	1.25	0.22-7.08	1	0.962	0.24-3.89	1
ERPR-/HER2+ Vs ERPR-/HER2-	0.444	0.09-2.28	0.428	0.909	0.21-4.02	1
ERPR+/HER2+ Vs ERPR+/HER2-	1.591	0.34-7.37	0.703	0.49	0.13-1.79	0.348
ERPR-/HER2+ Vs ERPR+/HER2-	0.606	0.15-2.49	0.728	0.424	0.11-1.69	0.322
ERPR-/HER2+ Vs ERPR+/HER2+	0.356	0.06-2.08	0.384	0.582	0.12-2.88	0.689

LN, lymph node; CI, confidence interval; ER, estrogen receptor; PR, progesterone receptor; HER2, human epidermal growth factor receptor 2.

## DISCUSSION

In the present study we report various clinicopathological characteristics of BC and their association with nodal status in this cohort of 150 BC patients from Karachi, Pakistan. The results for ‘1-3’ LN involvement were not significant while age (menopausal status), tumor grade and stage, tumor size, LVI, PNI and skin and nipple invasion showed significant risk prediction for ‘>3’ LNs involvement. Western studies have previously evaluated axillary nodal status with clinicopathological variables like age, histologic grade, tumor stage, receptor status and LVI.^7^,^11^

Axillary LN status is an important predictor for BC prognosis. Rates of BC recurrence are higher for patients with LN metastasis (70% risk) as compared to patients diagnosed with LN negative BC (20% - 30% risk). Mammon et al.^10^ showed 25% cases as LN negative, 35.6% with 1-3 LNs involved and more than 60% with >4 LNs involved. Aziz-un-Nisa et al.^3^ reported 28.7% of cases with no nodal involvement, 23.3% of cases with 1-3 positive LNs and 48% with >3 LNs involved. Our study is in agreement with findings of these researchers with 24.7% LN negative, 31.3% with 1-3 LNs and 44% with >3 positive LNs.

Local studies have reported younger age, higher grade and stage at the time of presentation.^1^,^2^ We also report similar demographic findings as maximum number of high grade (II & III; 92%) and late stage (II & III; 83%) tumors with nodal metastasis were also seen in our patients with mean age of 46.7 years. Age of BC is 40 to 50 years in the developing countries (including Pakistan) at time of presentation, with a mean of 47/48 years 2,10 and 60 to 70 years in the developed world with a mean of 61 to 63 years.^14^,^15^

An inverse relationship between age and axillary LN involvement has been reported previously with younger patients (<40 years) having a higher risk for nodal involvement.^13^ In our study a statistically significant association was observed between age and LN involvement (‘>3’ LN) with a lower risk for postmenopausal women (OR 0.33; 95% CI 0.14-0.76; p < 0.013). However two studies done in Asian populations showed no relationship with age.^9^,^12^

Infiltrating ductal carcinoma (IDC) was found to be the most common histological type of BC (93.3%) in our study and other local studies as well.^1^,^2^,^10^ In contrast developed countries have a frequency of 70% to 80% IDC and 5% to 15% of ILC.^16^ Lower incidence of ILC in our patients may be attributed to multiple factors that have known protective effect like high parity, birth of first child at an early age, prolonged lactation etc.^16^

Scarff-Bloom-Richardson histological grades reported in literature include 5.1%-33% for grade I, 31%-57% for grade II and 20%-63.6% for grade III.^2^,^7^ Mammon et al.^10^ reported 94.7% high grade tumors (grade II and III) in their study. Distribution of histological grades in our study (92% grade II & II) is within the ranges reported in literature, with a lower risk of nodal metastasis for grade I as compared to grade II (OR 0.88) and grade III tumors (OR 0.77).

Late presentation of BC in our country has been highlighted by different researchers.^1^,^10^,^17^ Naeem et al.^1^ reported 60% of patients in stages III and IV, while Gilani et al.^17^ reported figures of 71% and 63% respectively from two different cancer hospitals in Lahore, Pakistan. Similar findings have been reported by other researchers.^2^ We also report 69.4% patients in stages III and IV (p = 0.0001). The highest risk of nodal metastasis was seen in stage IV (OR 36) followed by 9-fold risk for ‘1-3’ LNs and a 24-fold risk for ‘>3’ LN in stage III tumors.

Larger tumor size at the time of presentation is associated with an advanced stage. In a comparison of BC cases over the last three decades at AFIP, there were 30.6% patients with tumor >5 cm in size, and 18.8% with tumor < 2 cm.^10^ Large tumor size is a surrogate marker for nodal metastasis and hence poor prognosis. Most of the studies have reported that BC patients with tumors > 2 cm or larger had a higher risk of nodal metastasis,^13^,^14^ while some studies did not report similar findings.^18^,^19^ We also report a statistically significant (p < 0.001) association between tumor size and lymph node involvement with high risk of nodal metastasis for tumors 2 – 5 cm or > 5 cm in size (Table-II).

LVI has been reported as a predictor of nodal metastasis in various studies.^9^,^13^ In a study on Chinese BC patients, He et al.^9^ reported an association between tumor grade, stage and LVI with nodal status. Our results showed a higher frequency of LVI (35.5%) with a high risk of nodal metastasis in these patients (OR 1.8 for ‘1-3’ LN & 5.8 for ‘>3’ LNs). High grade advanced breast cancers can present with skin involvement and Paget’s disease as reported by Mamoon et al.^10^ (36% cases presenting with nipple and skin invasion). The present study reports 32.7% cases of nipple and skin invasion with statistically significant association with nodal status (p <0.05) and higher risk for metastasis to the LNs (OR 3.14 for ‘1-3’ LN & 4 for ‘>3’ LNs).

Previously published data regarding association between ER, PR and Her2/neu reactivity and nodal status gives conflicting reports. Bevilacqua et al.^20^ found increased frequency of LN involvement in ER/PR positive cases while Moosavi et al.^13^ and Friedman et al.^14^ found no statistically significant association between ER/PR/Her2 reactivity and nodal status. Our observations were similar to the latter two studies with no relationship between these two variables. Similarly a study done in Pakistan also showed no significant correlation with lymph node metastasis.^3^

## CONCLUSION

Our study confirms BC as a heterogeneous disease with varied clinical, pathological and molecular features, and prognostic behavior. Results of this study reveal statistically significant differences when clinicopathological characteristics were compared with nodal status. However larger prospective studies are required to develop and validate a nomogram for prediction of nodal metastasis in our population.
